# DRIMust: a web server for discovering rank imbalanced motifs using suffix trees

**DOI:** 10.1093/nar/gkt407

**Published:** 2013-05-17

**Authors:** Limor Leibovich, Inbal Paz, Zohar Yakhini, Yael Mandel-Gutfreund

**Affiliations:** ^1^Department of Computer Science, Technion - Israel Institute of Technology, Technion City, Haifa 32000, Israel, ^2^Department of Biology, Technion - Israel Institute of Technology, Technion City, Haifa 32000, Israel and ^3^Agilent Laboratories Israel, 94 Em Hamoshavot Road, 49527 Petach-Tikva, Israel

## Abstract

Cellular regulation mechanisms that involve proteins and other active molecules interacting with specific targets often involve the recognition of sequence patterns. Short sequence elements on DNA, RNA and proteins play a central role in mediating such molecular recognition events. Studies that focus on measuring and investigating sequence-based recognition processes make use of statistical and computational tools that support the identification and understanding of sequence motifs. We present a new web application, named DRIMust, freely accessible through the website http://drimust.technion.ac.il for *de novo* motif discovery services. The DRIMust algorithm is based on the minimum hypergeometric statistical framework and uses suffix trees for an efficient enumeration of motif candidates. DRIMust takes as input ranked lists of sequences in FASTA format and returns motifs that are over-represented at the top of the list, where the determination of the threshold that defines top is data driven. The resulting motifs are presented individually with an accurate *P-*value indication and as a Position Specific Scoring Matrix. Comparing DRIMust with other state-of-the-art tools demonstrated significant advantage to DRIMust, both in result accuracy and in short running times. Overall, DRIMust is unique in combining efficient search on large ranked lists with rigorous *P*-value assessment for the detected motifs.

## INTRODUCTION

The study of sequence elements that enable molecular recognition in a variety of cellular processes is an important component in improving our understanding of regulation in living cells. Transcription factor (TF) activity, for example, often depends on the identification of specific targets using molecular pattern recognition mechanisms that involve sequence motifs. Sequence recognition plays a role in other molecular levels, as well. The occurrence of short binding motifs in RNA molecules plays a central role in enabling controlled regulation by RNA-binding proteins (RBPs) and by microRNAs. For example, the Pumilio family proteins regulate target messenger RNAs by recognizing and binding sequence elements in 3′ untranslated regions (UTRs) (1). Protein modification and protein–protein interactions are also potentially driven by mechanisms that involve specific protein sequence recognition such as the phosphate-binding loop (2,3).

Studies using techniques such as ChIP-chip (4), ChIP-PET (5), ChIP-seq (6) and ChIP-exo (7) lead to genome-wide measurement data pertaining to the TF binding affinity of various genomic regions, obtained in actual samples and in several conditions. Similarly, messenger RNA targets of RBPs are studied using techniques like RNA immunoprecipitation (RIP)-chip (8), crosslinking and immunoprecipitation (CLIP) (9) and photoactivatable-ribonucleoside-enhanced crosslinking and immunoprecipitation (PAR-CLIP) (10). Stable isotope labeling by/with amino acids in cell culture (SILAC) (11) and other proteomic techniques can be used to characterize the effect of amino acid sequences on protein function. Computational tools and approaches to motif discovery form part of the data analysis workflow that is used to extract knowledge and understanding from this type of studies. Motif discovery has attracted much research interest in recent years, resulting in more than a hundred different tools for motif discovery (12,13). A large subset of motif finders such as Multiple EM for Motif Elicitation (MEME) (14), NMica (15), AlignACE (16), MDscan (17), Yeast Motif Finder (YMF) (18), Gapless Local Alignment of Multiple sequences (19) and Suite for Computational identification Of Promoter Elements (SCOPE) (20) fit position weight matrices to the sequence data. Recently, efficient motif discovery tools were designed to handle large sets of data arising from the aforementioned high-throughput measurement techniques, as for example MEME-ChIP (21), Discriminative Regular Expression Motif Elicitation (DREME) (22) and XXmotif (23). Most techniques are designed to find motifs by seeking elements that occur more often than expected in a set of sequences. Many of these techniques compare a target set with a background set, such as in XXmotif (23). It is often the case, however, in biological measurement data, that results are given as a ranked list of quantities. Such is the case for data generated by ChIP-seq or CLIP, for example. Statistical approaches such as Gene Set Enrichment Analysis (24) and minimum hypergeometric (mHG) (25–28) address motif enrichment in ranked lists of elements. We have previously developed DRIM (25), a motif-finding approach that exploits the ranking derived from experimental measurements to discover k-mers that are rank imbalanced in the input list based on the mHG statistics. The search for rank imbalanced motifs allows for much more flexibility and is therefore more compatible with the character of the actual measurement results. The mHG model allows for a rigorous statistical assessment of the results without the need to run simulations. To overcome the computational challenges associated with large motif searches, we have recently developed the DRIMust algorithm (29). The algorithm is based on suffix trees, an approach also suggested in (30–32). DRIMust allows for an efficient enumeration of motif candidates, which are then assessed using the mHG statistics. Tree-based approaches have been previously efficiently used in other motif search algorithms, such as the beam search algorithm, which is an enumerative algorithm for identifying enriched cis-regulatory elements in sets of commonly regulated genes (33).

In this work, we introduce the DRIMust web server and describe its utility in supporting the search of rank imbalanced motifs. DRIMust takes as input ranked lists of sequences in FASTA format and returns motifs that are over-represented at the top of the list, where the determination of the threshold that defines top is data driven. In cases where sequence ranking is not relevant or not available, DRIMust allows the user to upload positive and negative sets of sequences. In the latter case, DRIMust will search for enriched motifs in the positive set using the negative set as the background. DRIMust is efficient and thus allows searching in large data sets, searching for long motifs as well as searching motifs over large alphabets in short running times. The resulting motifs are presented as a Position Specific Scoring Matrix (PSSM) in a graphical WebLogo format; the matrix can also be downloaded as a text file. For every motif, a *P*-value is indicated. DRIMust is freely accessible through the website http://drimust.technion.ac.il/.

## DRIMUST METHODOLOGY

The DRIMust approach seeks rank imbalanced motifs, given a ranked list of sequences 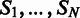. Rank imbalanced motifs are substrings that appear more often at the top of the list compared with the remainder of the list. Eden *et al.* (25) described the mHG statistics used for the assessment of rank imbalanced motifs. A unique feature of the mHG statistics is that the cutoff between the top and the rest of the list is determined in a data-driven manner so as to maximize motif enrichment. This is done by computing the motif enrichment over all possible set partitions and identifying the cutoff at which maximal statistical significance is observed. The algorithmic approach of DRIMust is based on suffix trees, allowing efficient enumeration of the motif search space (29).

### Enrichment analysis using mHG statistics

We have previously described an algorithm to identify the enrichment of a set of genes, *A*, in a ranked list of genes, using mHG statistics (25). Given a total number of genes *N*, with *B* of these genes belonging to *A*, and *n* of these genes being in the target set (e.g. differentially expressed genes), the probability that *b* or more genes from the target set are also in *A* is given by the tail of a hypergeometric random variable (HGT):

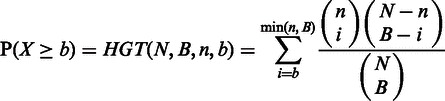



For a ranked genes list 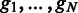, we define a label vector 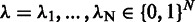 according to the association of the ranked genes to *A*, that is, 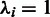 if and only if 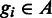. The mHG score is then defined as 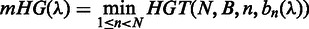, where 
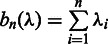
.

In other words, the mHG score is the optimal HGT probability that is found over all possible partitions induced by the ranking. As such, this score must be corrected for multiple testing. A dynamic programming algorithm for computing the exact *P-*value of a given mHG score is described in (25). More specifically, given a ranked list of genes, a subset *A*, and a corresponding mHG score *s*, the mHG *P*-value tells us the exact probability of observing an mHG score *s′* ≤ *s* under the null assumption that all occurrence configurations of *A* in the ranked list are equiprobable. In practice, DRIMust uses Stirling’s approximation (34) to compute all binomial coefficients needed to assess HGTs.

### Suffix trees

A suffix tree is a data structure that represents all the suffixes of a given string in a way that allows fast implementation of many string operations. A path from the root to a leaf in the tree represents a suffix. Each leaf of the tree holds information about the indices of strings that contain the suffix, and the starting positions of this suffix within each such string. Restoring all occurrences of a suffix is thus enabled, which further allows for the detection of DNA, RNA or protein substrings that manifest a significant occurrence pattern in a set of biologically related sequences. There are several algorithmic approaches to the efficient construction of a suffix tree for a collection 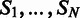 of strings (35–37). DRIMust uses a version that takes *O(M)* time, where 
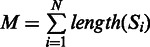
 by implementing Ukkonen’s algorithm for generalized suffix trees construction (37).

In DRIMust, an initial motif search phase produces k-mers, which are words over the alphabet of the input sequences. These candidate k-mers are derived by enumerating paths of length *k* in the generalized suffix tree generated for the input sequences. Next, the statistical significance of the k-mers is calculated using the mHG statistics [for more details on how *P*-values are computed in the nodes of the suffix tree, see (29)]. In the next stage, the promising k-mers are extended to produce PSSMs.

### PSSM extension

The promising k-mers are passed as input to a process that extends them to PSSMs. Extension is obtained by a heuristic approach based on the Hamming neighbors of the best 50 exact motifs. Briefly, starting from a single k-mer, Hamming neighbors (of length *k*) are added to a set of motifs as long as the PSSM representing that set improves the observed enrichment *P*-value.

## DRIMUST DESCRIPTION

### Input

**Figure 1.**
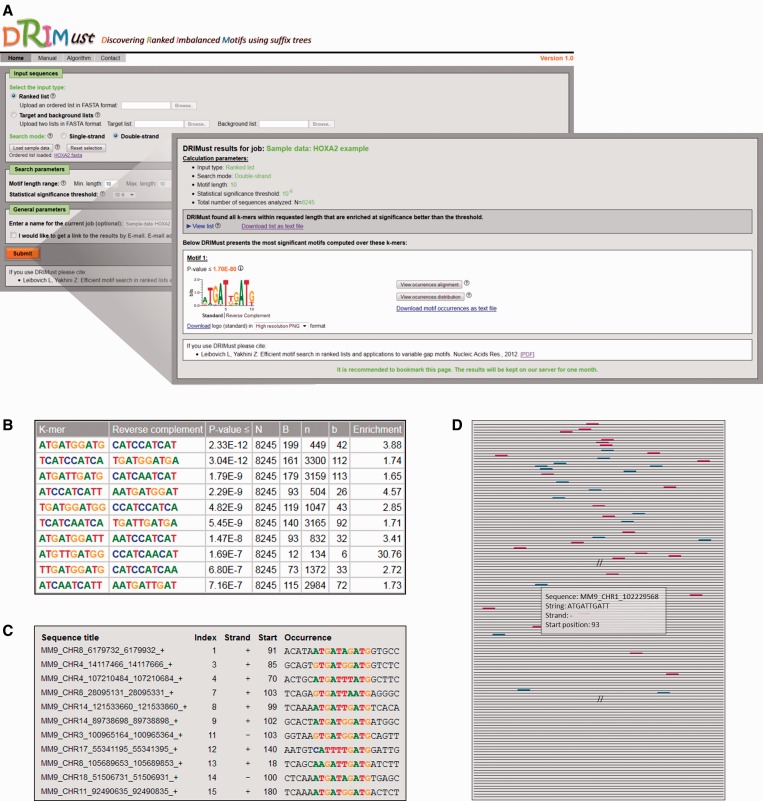
A view of DRIMust input and output pages. We ran DRIMust on the HOXA2-binding regions from the ChIP-seq experiment by Donaldson *et al.* (38). In this data set, the DNA sequences were ranked according to their binding *P*-values (as defined by Donaldson *et al.*). DRIMust was run using the double-strand search mode, and the rest of the parameters were set to default. The full data set is provided as an example in the manual page of DRIMust web server. (**A**) When clicking the submit button (bottom left), an output page, summarizing the best motifs found, is shown to the user. (**B**) By clicking the ‘view list’ button, the user is provided with a list of the significant k-mers and the statistical details of each motif. (**C**) By clicking the ‘view occurrences alignment’ button, the user is provided with an aligned list of motif occurrences mapped into the input sequences. (**D**) By clicking the ‘view occurrences distribution’ button, a window depicting the occurrences of the motif in the query sequences is opened. More details on each occurrence are shown when placing the cursor on the occurrence box.

DRIMust is designed to search for enrichment of motifs in large datasets of DNA, RNA or protein sequences (up to 40 000 sequences and up to 4 000 000 characters), which can be represented as ranked lists or as two separated sets of targets and background. Ranking should be provided by the user according to the research question of interest, e.g. binding affinity for ChIP-seq data. In the case of uploading target and background sets, the latter can be a selected random set of sequences taken from the genome. When uploading the input data, the user is prompted to choose between submitting one ranked list of sequences in FASTA format or two sets of target and background (see Figure 1A). In both options, the user is prompted to choose the preferable search mode: single-strand (suitable, though not restricted, to RNA) or double-strand for DNA sequences. The default query type is single-strand search mode. In the double-strand mode, DRIMust searches for motifs consisting of a sequence and its reverse complement that are enriched at the top of the input list. As described earlier in the text, in all search modes, DRIMust searches for motifs that are over-represented at the top of the ranked list of sequences, where the determination of the threshold that defines top is data driven (see ‘DRIMust Methodology’ section).

DRIMust allows searching for k-mers of a specific length or in a range of lengths. The default range is 5–10 characters in single-strand mode and 10 characters in double-strand mode, whereas the maximal length range supported by the web server is 4–20 characters. Notably, when a range of lengths is provided, DRIMust will search for the most significant motif, which will not necessarily be the longest one. When a certain motif length is expected, the user is encouraged to define an exact length. Next, the user can choose to change the default statistical significance threshold (set to 10^−^^6^) to any threshold between 10^−^^2^ and 10^−^^15^. Finally, although not required, DRIMust supports including an e-mail address to which the results will be automatically sent when the analysis is completed. This option is useful when sending long jobs. After uploading the input data and defining the parameters, the users are prompted to submit their job.

### Output

DRIMust motif-searching process is divided into two phases. In the first phase, DRIMust searches for k-mers, which are over-represented at the top of the input ranked list of sequences. As a default, DRIMust will report enriched k-mers having *P*-value better than the selected stringency. In the second phase, DRIMust expands the most promising k-mers heuristically and creates motifs represented by PSSMs. An average job for data sets containing 4000 DNA sequences, total 2 000 000 characters, takes 1 min and 10 s when double-strand search mode is used and 15 s when single-strand search mode is used.

We ran DRIMust on the HOXA2-binding regions from the ChIP-seq experiment by Donaldson *et al.* (38). In this data set, the DNA sequences were ranked according to their binding *P*-values [as defined by (38)]. As demonstrated in Figure 1A, the best motifs are presented in the output page as PSSMs displayed in a graphical WebLogo representation (39) and also provided as a downloadable text files. The *P*-value of each motif is indicated above the logo. Furthermore, the user is provided with a detailed list of all significant k-mers that DRIMust has found to be enriched at significance level better than the threshold (Figure 1B). In addition, each row includes information about the total number of input sequences (*N*); the total number of sequences containing the motif (*B*); the index that is selected by the mHG statistics as the division of the input list into target and background (*n*)—which optimizes the enrichment of the motif at the top *n* sequences of the list; and the number of sequences containing the motif amongst the top *n* sequences (*b*). Finally, the enrichment value, which compares the abundance of the motif at the top of the list to the abundance at the entire list, defined by (*b*/*n*)/(*B*/*N*), is indicated. When a double-strand search mode is chosen, the reverse complement motif is also shown. Clicking the ‘View occurrences alignment’ button (shown in Figure 1A) opens up a window containing an aligned list of the motif occurrences mapped to the input sequences (Figure 1C). In addition, clicking the ‘View occurrences distribution’ button (which is also shown in Figure 1A) depicts the occurrences of the motif in the query sequences schematically (Figure 1D). This presentation nicely demonstrates the rank imbalanced representation of the motif in the ranked list. Furthermore, detailed information about each occurrence can be obtained by placing the cursor on a colored box (representing a motif occurrence).

## RESULTS AND DISCUSSION

In recent years, high-throughput binding techniques have been developed [e.g. ChIP-seq (4) for protein–DNA, PAR-CLIP (10) for protein–RNA and SILAC (11) for protein–proteins]. These methods yield extensive lists of potential targets, ranked according to their binding affinity. The main advantage of our method, implemented in DRIMust, is that it searches for enriched motifs in the entire ranked list and does not require defining a fixed set of sequences as in the case of other motif-search algorithms such as MEME (14), PhyloGibbs (40) and others. Nevertheless, DRIMust does provide the option of uploading a target and background sets predefined by the user. In the latter case, DRIMust searches motifs that are overrepresented at the target set compared with the background set. To evaluate the performance of DRIMust in comparison with other state-of-the-art methods, we ran DRIMust on 24 examples generated from high-throughput binding experiments—10 TFs and 14 RBPs—and compared the results with those obtained by using four other methods: the standard MEME program (14); the DREME program (22) from the MEME suit (http://meme.nbcr.net), which was optimized for fast analysis of large data sets; XXmotif (23), a recent web server, which was designed for efficient extraction of position weight matrices from large datasets; and SCOPE (20), which was designed to identify candidate regulatory DNA motifs from sets of genes that are coordinately regulated. Almost all the input examples comprised ranked lists, except for p53, which comprised target and background sets. As MEME, DREME and XXmotif expect a target set as input, we converted the ranked lists into target sets by taking the top 100 sequences in the case of MEME (restricted by MEME’s limitation of 60 000 characters) and the top 20% sequences for the other tools. The results of the comparison are summarized in Supplementary Table S1. As demonstrated, in 22 of the 24 test examples, DRIMust found the motifs that were compatible with the known motifs as the most significant result. In comparison, DREME found the known consensus in 19 cases, XXmotif detected the literature motif in 16 cases, whereas MEME and SCOPE detected the known motif in only half of the cases. Notably, in the other methods tested, the known motifs were not always reported as the best motif. Strikingly, while DRIMust was tested on the largest data sets, in all cases, DRIMust completed the computations faster than the other tools. As demonstrated in Supplementary Table S1, the longest job took 1 min and 21 s on DRIMust (for a data set containing 9995 sequences, each of length 100 nucleotides).

Overall, the web-application DRIMust has several advantages over existing methods. First, unlike many other approaches, it does not exhaustively search over all possible k-mers space and therefore can detect long motifs and motifs over large alphabets. DRIMust runs efficiently and allows for timely interaction with the results, through a friendly interface and a clear output format. Most importantly, by working with ranked lists, DRIMust avoids the arbitrary designation of fixed sets of sequences and exploits the ranking derived from experimental measurements. More than that, DRIMust uses the ranking to discriminate true motifs from other irrelevant sequence elements (such as AT repetitive elements that are abundant in 3′UTRs), as the latter are not correlated with the ranking and are therefore ignored by DRIMust. This explains the observed accuracy of DRIMust compared with other tools in many of the examples shown in Supplementary Table S1.

As biological techniques such as ChIP-seq (6), ChIP-exo (7), CLIP (9), PAR-CLIP (10) and others produce ranked lists, using DRIMust is the natural choice for motif discovery in these cases, as arises from the comparison in Supplementary Table S1. DRIMust can efficiently deal with the large data sets generated by such methods, making it preferable for large volume data. Nevertheless, DRIMust is also useful in cases when there are clear target and background sets. In the latter scenario, the enrichment is calculated using the hyper-geometric distribution.

## SUPPLEMENTARY DATA

Supplementary Data are available at NAR Online: Supplementary Table 1 and Supplementary References [4,10,38,41–44].
